# Effects of acute hypobaric hypoxia on thermoregulatory and circulatory responses during cold air exposure

**DOI:** 10.1186/s40101-020-00237-7

**Published:** 2020-09-10

**Authors:** Sora Shin, Yoshiki Yasukochi, Hitoshi Wakabayashi, Takafumi Maeda

**Affiliations:** 1 Graduate School of Design, Kyushu University, 4-9-1 Shiobaru, Minami-ku, Fukuoka, 815-8540 Japan; 2 Department of Human Functional Genomics, Advanced Science Research Promotion Center, Mie University, 1577 Kurima-machiya, Tsu, Mie 514-8507 Japan; 3 Faculty of Engineering, Hokkaido University, N13 W8, Kita-ku, Sapporo, Hokkaido 060-8628 Japan; 4 Department of Human Science, Faculty of Design, Kyushu University, 4-9-1 Shiobaru, Minami-ku, Fukuoka, 815-8540 Japan; 5 Physiological Anthropology Research Center, Faculty of Design, Kyushu University, 4-9-1 Shiobaru, Minami-ku, Fukuoka, 815-8540 Japan

**Keywords:** Cold stress, Altitude, Thermoregulation, Skin temperature, Individual differences

## Abstract

**Background:**

The thermoregulatory responses during simultaneous exposure to hypoxia and cold are not well understood owing to the opposite reactions of vasomotor tone in these two environments. Therefore, the purpose of this study was to investigate the influences of hypobaric hypoxia on various thermoregulatory responses, including skin blood flow (SkBF) during cold exposure.

**Methods:**

Ten subjects participated in two experimental conditions: normobaric normoxia with cold (NC, barometric pressure (P_B_) = 760 mmHg) and hypobaric hypoxia with cold (HC, P_B_ = 493 mmHg). The air temperature was maintained at 28 °C for 65 min and gradually decreased to 19 °C for both conditions. The total duration of the experiment was 135 min.

**Results:**

The saturation of percutaneous oxygen (SpO_2_) was maintained at 98–99% in NC condition, but decreased to around 84% in HC condition. The rectal and mean skin temperatures showed no significant differences between the conditions; however, the forehead temperature was higher in HC condition than in NC condition. The pulse rate increased in HC condition, and there was a strong negative relationship between SpO_2_ and pulse rate (*r* = − 0.860, *p* = 0.013). SkBF and blood pressure showed no significant differences between the two conditions.

**Conclusion:**

These results suggest that hypobaric hypoxia during cold exposure did not alter the overall thermoregulatory responses. However, hypobaric hypoxia did affect pulse rate regardless of cold exposure.

## Background

When the human body is exposed to cold, skin blood flow (SkBF) decreases to minimize the heat loss from the body to the environment [1]. This thermoregulatory adjustment changes skin temperature (T_sk_), causing it to decline. Further, if the heat storage cannot be maintained, rectal temperature (T_re_) decreases. However, the thermoregulatory responses during simultaneous exposure to hypobaric hypoxia and cold are rather equivocal. This stems from the fact that local tissue hypoxia elicits an increase in blood flow in order to maintain the usual oxygen delivery rate for sustained metabolism [2].

Previous studies have shown the changes in T_re_ and mean T_sk_ during simultaneous exposure to hypoxia and cold. Fukazawa et al. [3] reported no significant differences on T_re_, but higher mean T_sk_ at 17 °C and a simulated altitude of 5000 m compared to normobaric normoxia. Blatteis and Lutherer [4] also found the same results at 10 °C under two different altitudes (3350 and 4340 m). In another study, Cipriano and Goldman [5] conducted experiments at three different air temperature (T_air_) (15.5, 21, and 26.5 °C) and two different simulated altitudes (2500 and 5000 m). They found lower T_re_ at 15.5 °C, but no differences in T_re_ at T_air_ 21 and 26.5 °C at both altitudes compared to sea level. Higher mean T_sk_ was observed when the T_air_ was 15.5, 21 °C, and has an altitude of 5000 m compared to sea level. When the T_air_ was 26.5 °C, no differences on T_re_ and mean T_sk_ were found at both altitudes compared to sea level.

Some studies have reported regionally different vascular reactions based on changes in T_sk_ during hypoxia and thermoneutral environments [6] and increased forearm SkBF during hypoxic exposure in thermoneutral environments [7, 8]. However, very little research has been conducted on the effects of simultaneous exposure to hypoxia and cold on local responses. Investigating local thermoregulatory responses would help better understanding of physiological responses at high altitudes.

Individual differences should also be noted when investigating physiological responses in hypoxia. Brown et al. [9] have reported that certain subjects showed significantly different T_sk_ between hypoxic cold and normoxic cold environments while others did not. Also, previous studies have found genetic polymorphisms that can affect saturation of percutaneous oxygen (SpO_2_) responses in hypoxia and polymorphisms with susceptibility to high-altitude pulmonary edema [10, 11].

Therefore, this study aimed to shed light on the effects of hypobaric hypoxia on overall and local thermoregulatory responses during cold exposure and investige individual differences of physiological responses. We hypothesized that (1) regionally different vascular reactions would be observed in hypobaric hypoxia during cold exposure and (2) SpO_2_ would be different among individuals.

## Methods

### Subjects

Ten male university students participated in this study (mean ± standard deviation [SD] age 22.7 ± 1.9 years; height 174.8 ± 5.9 cm; body mass 65.3 ± 7.7 kg). All participants were free of cardiovascular, respiratory, and ear diseases. The subjects abstained from alcohol drinking, smoking, and strenuous exercise for the previous 24 h and were prohibited from taking any food and caffeine for 2 h prior to their scheduled tests. Written informed consent was obtained from all participants prior to their participation in this study. This research was approved by the Ethics Committee of the Faculty of Design, Kyushu University (Approval number 269).

### Experimental design and procedures

**Fig. 1:**
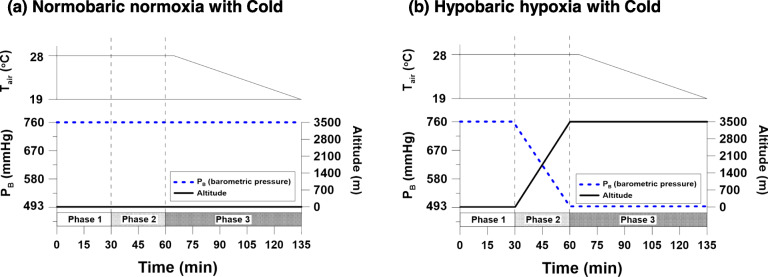
Experiment conditions of this study. (**a)** Normobaric normoxia with cold (NC); (**b)** hypobaric hypoxia with cold (HC). The experiment duration was divided into three phases. Phase 1 (P1) T_air_ was 28 °C and P_B_ was 760 mmHg in both conditions. Phase 2 (P2) T_air_ was 28 °C in both conditions, and while P_B_ was maintained at 760 mmHg in the NC condition, P_B_ was decreased from 760 to 493 mmHg in the HC condition. Finally during phase 3 (P3), T_air_ was decreased from 28 to 19 C in both conditions and P_B_ was 760 mmHg in NC and 493 mmHg in HC

All subjects participated in two experimental conditions: normobaric normoxia with cold (NC) and hypobaric hypoxia with cold (HC). The experimental conditions were randomly distributed, and each condition of a subject was separated by at least 72 h. The subjects wore only undershorts and short-sleeve T-shirts (0.13 clo) and they were maintained in a supine position. The total duration of the experiment was 135 min. In both NC and HC conditions, the T_air_ was maintained at 28 °C for 65 min and decreased to 19 °C for 70 min. Humidity was maintained at 50% RH in both conditions. In NC condition, normobaric normoxia (barometric pressure (P_B_) of 762.0 ± 2.9 (mean ± SD) mmHg; ≈ sea level) was maintained during the entire experiment. In HC condition, P_B_ was maintained at 764.9 ± 3.7 mmHg for 30 min and gradually changed to a hypobaric hypoxia environment (P_B_ of 493.0 ± 1.5 mmHg; ≈ 3500 m altitude) for 30 min, which was maintained until the end of the experiment. We divided the experiment duration into three phases. Phase 1 (P1) T_air_ was 28 °C and P_B_ was 760 mmHg in both conditions. Phase 2 (P2) T_air_ was 28 °C in both conditions, and while P_B_ was maintained at 760 mmHg in the NC condition, P_B_ was decreased from 760 to 493 mmHg in the HC condition. Finally during phase 3 (P3), T_air_ was decreased from 28 to 19 °C in both conditions and P_B_ was 760 mmHg in NC and 493 mmHg in HC (Fig. 1).

### Measurements

During the entire trial, T_re_ and T_sk_ were recorded every 5 s using a data logger (LT-8A, Gram Corporation, Japan). The T_re_ was measured using a thermistor probe that was inserted 13 cm beyond the anal sphincter of the rectum. The T_sk_ was measured by attaching thermistors to the skin corresponding to the following body regions with surgical tape: forehead, chest, forearm, hand, thigh, calf, and instep. Mean T_sk_ was estimated using a modified Hardy and DuBois’ equation: mean T_sk_ = 0.07 (T_forehead_) + 0.35 (T_chest_) + 0.14 (T_forearm_) + 0.05 (T_hand_) + 0.19 (T_thigh_) + 0.13 (T_calf_) + 0.07 (T_foot_) [12]. SkBF on the right proximal third of forearm and the right middle finger pad were measured using a laser doppler flowmeter (Advance Laser Flowmeter ALF21, Advance Company, Ltd., Japan). Pulse rate (PR) and SpO_2_ were monitored on an earlobe using the pulse CO-Oximetry (Radical-7, Masimo, USA) every 10 s. Blood pressure was measured every 15 min (UA-772K, A&D Medical, Japan). Shivering was evaluated by subjective responses. The subjects were given a button and were instructed to press the button whenever they felt shivering.

### Data analysis

The T_air_ changing rate was 0.15 and 0.13 °C min^− 1^ in NC and HC conditions, respectively. To minimize the effect of the different T_air_ changing rates, we conducted a data analysis according to the T_air_ changes instead of the time changes. Since the T_air_ was maintained at 28 °C during P1 and P2, the average values of 0–30 min and 30–60 min were used as the representative values of P1 and P2, respectively. For the data of P3, the average values for every 1 °C change in T_air_ were used. The SkBF values were presented as a percentage of P1. The differences between P1 and P3 of 19–20 °C were calculated for each condition and expressed as delta values (Δ). Statistical analyses were performed using SPSS v. 23.0 (IBM SPSS Statistics, USA). A two-way repeated measures ANOVA was run to determine the effect of different conditions over T_air_ changes. There was sphericity for the interaction term, as assessed by Mauchly’s test of sphericity (*p* > 0.05). One-way repeated measures ANOVA with a Bonferroni-adjusted post hoc test was used to establish significant differences over T_air_ within a condition. Paired *t* test was used to compare the difference between NC and HC conditions. A Pearson’s product-moment correlation was run to assess the relationship between physiological responses. Preliminary analyses showed the relationship to be linear with both variables normally distributed, as assessed by Shapiro-Wilk’s test (*p* > 0.05). The significance level was set at *p* < 0.05. All data were expressed as mean ± standard deviation.

## Results

### Rectal and skin temperatures

**Fig. 2:**
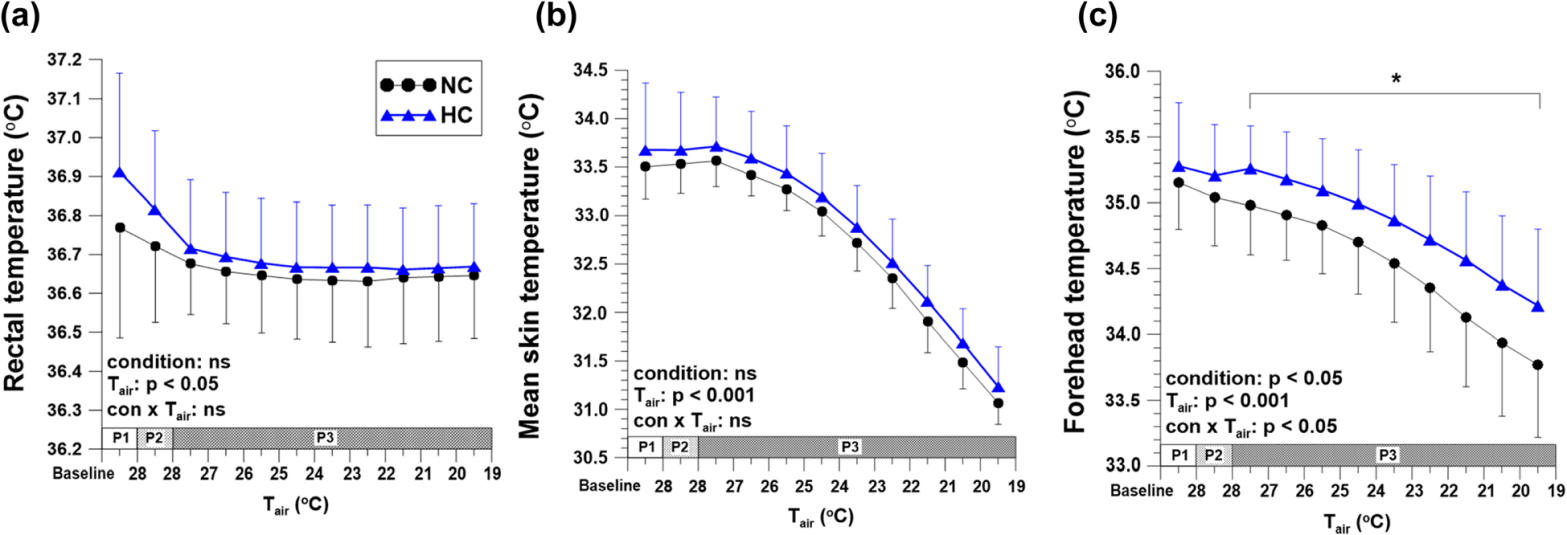
Rectal temperature (**a**), mean skin temperature (**b**), and forehead temperature (**c**) by air temperature changes in NC and HC. Data were expressed as mean ± SD. *Significant difference compared between conditions. NC normobaric normoxia with cold; HC hypobaric hypoxia with cold; phase 1 (P1) T_air_ was 28 °C and P_B_ was 760 mmHg in both conditions. Phase 2 (P2) T_air_ was 28 °C in both conditions, and while P_B_ was maintained at 760 mmHg in the NC condition, P_B_ was decreased from 760 to 493 mmHg in the HC condition. Finally during phase 3 (P3), T_air_ was decreased from 28 to 19 °C in both conditions and P_B_ was 760 mmHg in NC and 493 mmHg in HC

There were no significant differences in T_re_ and mean T_sk_ during the entire experiment between NC and HC conditions (Fig. 2a, b). Forehead temperature in HC condition was significantly higher than in NC condition at P3, and the difference between the conditions was 0.28–0.44 °C (*p* = 0.011) (Fig. 2c). The other T_sk_s showed no differences during the entire experiment.

### SpO_2_

**Fig. 3:**
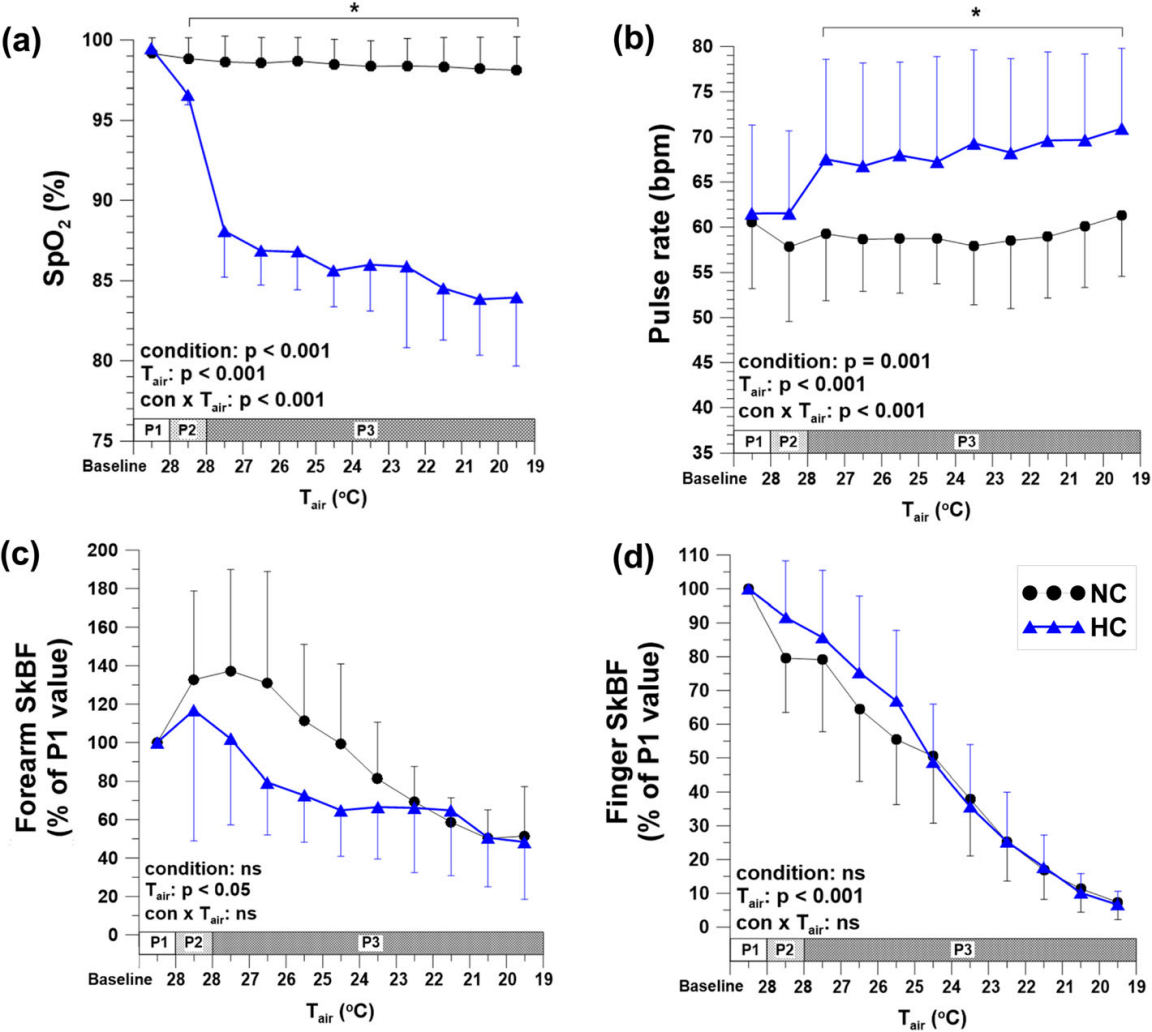
SpO_2_ (**a**), pulse rate (**b**), forearm SkBF (**c**), and finger SkBF (**d**) by air temperature changes in NC and HC. Data were expressed as mean ± SD. The SkBF values are presented as a percentage of P1. *Significant difference compared between conditions. NC normobaric normoxia with cold; HC hypobaric hypoxia with cold; SpO_2_ saturation of percutaneous oxygen; SkBF skin blood flow; phase 1 (P1) T_air_ was 28 °C and P_B_ was 760 mmHg in both conditions. Phase 2 (P2) T_air_ was 28 °C in both conditions, and while P_B_ was maintained at 760 mmHg in the NC condition, P_B_ was decreased from 760 to 493 mmHg in the HC condition. Finally during phase 3 (P3), T_air_ was decreased from 28 to 19 °C in both conditions and P_B_ was 760 mmHg in NC and 493 mmHg in HC

SpO_2_ was maintained at around 98–99% in NC condition during the entire experiment. In HC condition, SpO_2_ was 99.5 ± 0.4% at P1 and significantly decreased to 84.0 ± 4.3% at the end of the experiment (*p* < 0.05) (Fig. 3a).

### Pulse rate

There were no significant differences between two conditions at P1 and P2, respectively. PR in HC condition increased around 9.4 ± 4.4 bpm (*p* < 0.05), and it was significantly higher than NC condition at P3 (*p* < 0.05) (Fig. 3b).

### Skin blood flow 

SkBF on the forearm and the finger gradually decreased as the T_air_ decreased in both conditions. There were no significant differences in SkBF both on the forearm and finger between NC and HC conditions during the entire experiment (Fig. 3c, d).

### Blood pressure

There were no significant differences between NC and HC conditions in systolic blood pressure, diastolic blood pressure, and mean arterial pressure, respectively (Table 1).

**Table 1:** Mean arterial pressure (MAP) of the NC and the HC conditions

	Time (min)							
MAP (mmHg)	30	45	60	75	90	105	120	135
NC	79 ± 7	80 ± 5	80 ± 5	80 ± 5	83 ± 5	87 ± 2	89 ± 3	92 ± 6
HC	82 ± 6	79 ± 4	81 ± 7	83 ± 4	84 ± 5	86 ± 6	88 ± 7	90 ± 5
*p* value	ns	ns	ns	ns	ns	ns	ns	ns

*NC* normobaric normoxia with cold, *HC* hypobaric hypoxia with cold

### Subjects perceived shivering

There were no significant differences in the total number of perceived shivering between NC (9.7 ± 13.6) and HC (8.6 ± 10.3) conditions (*p* = 0.838). 

### Relationships among physiological responses

**Fig. 4:**
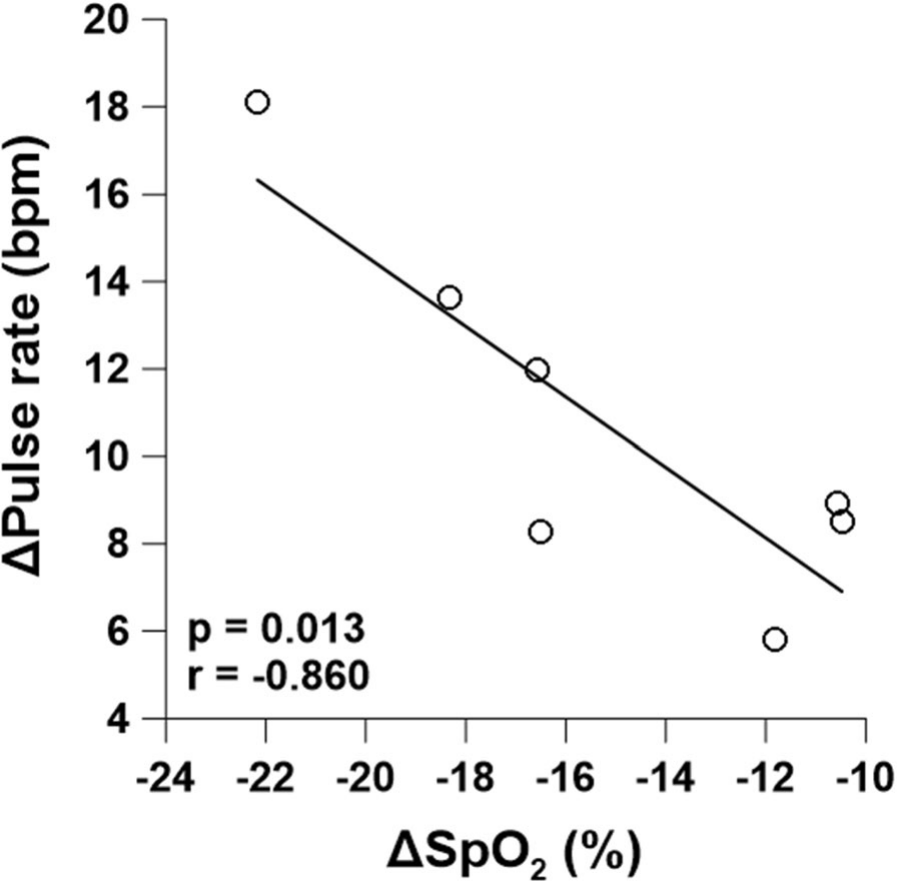
Relationships between ΔSpO_2_ and ΔPR in hypobaric hypoxia during cold exposure. The differences between P1 and P3 of 19–20 C were calculated for each condition and expressed as delta values (Δ)

There was a statistically significant, strong negative correlation between ΔSpO_2_ and ΔPR (*r* = − 0.860, *p* = 0.013). Subjects who showed a larger decrease in SpO_2_ had a larger increase in PR at simulated altitude of 3500 m (Fig. 4). However, there were no significant relationships for either ΔSpO_2_ or ΔPR between Δmean T_sk_, ΔT_re_, ΔT_forehead_, forearm SkBF, or blood pressure.

## Discussion

There were several principal findings in this study. First, T_re_ and mean T_sk_ in HC condition did not significantly differ from those in NC condition, suggesting that hypobaric hypoxia did not alter overall thermoregulatory responses in cold conditions. Second, the forehead temperature was significantly higher in HC compared to NC. And third, we found a negative relationship between ΔSpO_2_ and ΔPR, indicating that subjects more vulnerable to hypoxic environments demonstrated a greater burden on the heart.

### Rectal and mean skin temperature responses in hypoxia during cold exposure

Hypoxia is known to affect the cardiovascular system, increasing blood flow and heart rate in order to supply more oxygen to tissues [13]. Nevertheless, we detected no significant effects of hypoxia on SkBF and T_sk_ during cold exposure. There are at least two possible explanations for this observation. First, the levels of hypoxia might have been too mild to induce skin blood vasodilation. Second, hypoxia caused modest local vasodilation in the skin, but the effects of low T_air_ suppressed this response, making the overall reaction similar to that observed in normoxia. Either way, heat loss would be indifferent between the NC and the HC environments, suggesting that hypobaric hypoxia at the simulated altitude of 3500 m (493 mmHg) had no significant impact on the changes in T_re_ and mean T_sk_ during cold exposure.

### Forehead temperature in hypoxia during cold exposure

In this study, while other local T_sk_s were not altered by hypobaric hypoxia during cold exposure, forehead temperature was higher in HC condition than in NC condition. We were unable to unmask the related mechanisms in this study design. We assumed that this unique response may be related to skin blood vessels on the head or cerebral blood flow since they are anatomically close to each other. Further research is needed to identify the mechanisms related to changes in forehead temperature in hypoxia during cold exposure.

### Individual differences in SpO_2_ and pulse rate

A strong negative correlation (*r* = − 0.860) between ΔSpO_2_ and ΔPR was found in this study. The individual differences in SpO_2_ at high altitudes are well known, and it has been reported in previous studies [14–16]. These individual differences in SpO_2_ were explained as an aspect of difference in respiratory reflex and genetic polymorphisms [10, 17].

Penneys and Thomas [18] have reported a relationship between SpO_2_ with heart rate and blood pressure, respectively, in normobaric hypoxia without thermal stress. In this study, we only found a relationship between SpO_2_ and PR. We speculated that this was due to vasoconstriction during cold exposure. The combined results from the previous study and those from this study indicated that blood vessels were more sensitive to cold environments than hypoxia, but the heart was directly affected by SpO_2_ in hypoxia regardless of cold stress.

## Conclusion

We did not find any evidence that hypobaric hypoxia alters overall thermoregulatory responses, but higher forehead skin temperature was observed in hypoxia during cold condition. Also, a negative relationship between ΔSpO_2_ and ΔPR was found, indicating the subjects more vulnerable to hypoxic environments had a greater burden on the heart. These findings can be applied to alpine climbers and military personnel who are acutely exposed to hypobaric hypoxia under cold stress.

## Data Availability

The datasets used and/or analyzed during the current study are available from the corresponding authors on reasonable request.

## Abbreviations

NC: Normobaric normoxia with cold
HC: Hypobaric hypoxia with cold
P_B_: Barometric pressure
P1: Phase 1
P2: Phase 2
P3: Phase 3
SkBF: Skin blood flow
T_sk_: Skin temperature
T_re_: Rectal temperature
T_air_: Air temperature
SpO_2_: Saturation of percutaneous oxygen
MAP: Mean arterial pressure
